# Late-onset neuromyelitis optica spectrum disorder in AQP4-seropositivepatients in a Chinese population

**DOI:** 10.1186/s12883-015-0417-y

**Published:** 2015-09-04

**Authors:** Zhifeng Mao, Junjie Yin, Xiaonan Zhong, Zhihua Zhao, Wei Qiu, Zhengqi Lu, Xueqiang Hu

**Affiliations:** Multiple Sclerosis Center, Department of Neurology, The Third Affiliated Hospital of Sun Yat-Sen University, 600 Tianhe Road, Guangzhou, Guangdong 510630 China

## Abstract

**Background:**

Increasing rates of AQP4-seropositive neuromyelitis optica spectrum disorder (NMOSD) have been reported in late-onset patients (LONMOSD). However, the full range of clinical differences between early-onset and late-onset variants remain unclear. We describe the clinical features and outcomes of AQP4-seropositive LONMOSD patients in a Chinese population.

**Methods:**

This was a retrospective analysis of medical records in a cohort study of AQP4-seropositive NMOSD patients with early-onset (≤49 years) and late-onset (≥50 years) variants between January 2006 and February 2014. Demographic, clinical, neuroimaging and cerebrospinal fluid (CSF) findings and prognosis data were analyzed.

**Results:**

We identified thirty AQP4-seropositive LONMOSD patients (86.7 % women). The median age at onset was 57.5 years (range 50–70). There were similar onset frequencies between optic neuritis (ON) and longitudinally extensive transverse myelitis (LETM). Longer interval between (first) ON and LETM (median 13 vs. 4 months; *p* < 0.05), time from first symptoms to diagnosis of NMO (median 17 vs. 7 months, *p* < 0.05), higher comorbidities (66.7 vs. 26.7 %; *p* < 0.05), and more hypertension (26.7 vs.3.3 %; *p* < 0.05) were prevalent. NMO-like lesions were less common (10.7 vs. 41.6 %; *p* < 0.05), while the rate of non-specific lesions tended to be higher (53.6 vs. 29 %; *p* = 0.067). These patients displayed more severe Expanded Disability Status Scale (EDSS) in nadir (median 6.75vs.5; *p* < 0.05). Attacks often resulted in EDSS 4 within a short period (median 8 vs. 13.5 months; *p* < 0.05). At last follow-up, the EDSS score was more severe in these patients (median 5.25 vs. 4; *p* < 0.05). No significant predictors were identified.

**Conclusions:**

This study provides an overview of the clinical and paraclinical features of AQP4-seropositive LONMOSD patients in China and demonstrates a number of distinct disease characteristics in early vs. late onset. Older patients are more susceptible to disability in short course. However, these patients do not always display NMO-like lesions in the brain. Initial LETM may not necessarily be predominant as the initial symptom, contrary to previous reports. The higher comorbidities may warrant a modified approach of treatment.

## Background

Neuromyelitis optica (NMO) is an autoimmune inflammatory disease of the central nervous system (CNS) characterized by recurrent episodes of optic neuritis (ON) and longitudinally extensive transverse myelitis (LETM), in which autoantibodies against AQP4 play an important role [1–3]. In western countries, the disease mainly affects adults aged between 30 and 40 years [4].However, recent clinical studies suggest NMOSD in the elderly is not uncommon, possibly partly due to improved diagnostic methods in the aging population [5]. Multiple comorbidities may delay timely recognition of early symptoms of NMO/NMOSD and accurate diagnosis in the elderly (e.g. blurred vision may be attributed to macular degeneration). Additionally, a high occurrence of elevated AQP4 antibody titer has been reported in elderly patients, suggesting that there are even more unrecognized cases in this population [6, 7]. Moreover, it is unclear whether the early- and late-onset subgroups represent qualitatively distinct conditions or differences along a quantitative dimension, such as severity. The evolving epidemiology of NMO/NMOSD with an increasing prevalence in the elderly calls into question whether there is a difference between early-onset (EONMO/EONMOSD) and late-onset NMO/NMOSD (LONMO/LONMOSD) phenotypes in patients who are AQP4-seropositive, and warrants review as to whether a different treatment approach is appropriate. Furthermore, treatment decisions may be especially complicated in the elderly, given the more likely presence of multiple comorbidities and subsequent iatrogenic complications [8]. However, as far as we know, there have been few studies into the potential differences between AQP4-seropositive EONMO/NMOSD and LONMO/LONMOSD [9], and there is scarce epidemiological data from developing countries. Additionally, older patients tend to report decreased symptom severity in other autoimmune disorders [10, 11]. However, this phenomenon has not typically been supported in NMO studies [12]. Unfortunately, previous NMO studies have lacked appropriate controls (e.g. early onset group) to test this hypothesis [12]. Thus, there is inadequate conclusive evidence to confirm the greater severity of AQP4-seropositive NMO/NMOSD in late-onset patients.

In this study we report a detailed clinical analysis of 30 AQP4-seropositive NMO/LONMOSD patients ≥ 50 years old (age at onset of first symptoms) and compare the results with those of AQP4-seropositive EONMO/EONMOSD patients (age at onset of first symptoms ≤ 49 years), and we describe several novel features associated with this age group.

## Methods

### Patient selection and assessment

Thirty consecutive late-onset AQP4-seropositive NMO or NMOSD patients were identified from a clinical review chart retrospectively between January2006 and February2014. Among them, 23 AQP4-seropositiveNMO patients fulfilled the 2006 criteria [13], and 7 had either isolated AQP4-seropositive ON or AQP4-seropositive LETM [14]. For simplicity, we use the term NMOSD to encompass both NMO and NMOSD. Seropositivity for NMO-IgG was detected with an anti-AQP4 antibody assay on an anti-AQP4 transfected cell line from a commercial Biochip kit (Euroimmun, Lubeck, Germany) [15]. All patients tested positive for AQP4 antibodies at their earliest available sample. All patients must have had one or more follow-up visits at our center more than one year after the onset of symptoms. These patients were assessed by three neurologists in our multiple sclerosis (MS) clinic (XH, WQ and ZL).Clinical information was obtained by the authors or referring physicians at the acute stage of the disease. Follow-up information was obtained at regular intervals after symptom onset. Telephone interviews were typically used if the patients had been asymptomatic for years.

Data for missed study visits were obtainedby review of all available medical records. The Expanded Disability Status Scale (EDSS) scoring system was used to estimate disability during and after the onset episode, at the time of the first documented neurological examination, and at each visit [16]. The nadir EDSS score was determined as the maximum points during the acute episodes.

Patients who developed AQP4-seropositive NMOSD at an age ≥ 50 years (age at onset of first symptoms) were classified as those with LONMOSD and were considered cases. Early-onset patients (age at onset of first symptoms ≤ 49 years), matched for sex and disease duration, were randomly chosen for each case and were considered controls (or patients with EONMOSD). The cutoff age for early- verse late-onset AQP4-seropositive NMOSD was established based on previously published literature [12], and similar thresholds have been used in other autoimmune disorders, such as myasthenia gravis [10].

We collected the following variables: demographic, clinical, neuroimaging and cerebrospinal fluid (CSF) findings. Clinical variables included follow-up time (period from enrollment to last visit), total disease duration (the time elapsing from the date that the patient initially complained of symptoms of NMOSD up to the point of last visit), and comorbidities. Symptoms that occurred within one month after the initial symptoms of relapse were considered part of the same episode. Methylprednisolone administered intravenously was prescribed in the acute stage, and some patients received azathioprine or tacrolimus in the remission stage.

Other autoimmunity included coexisting autoimmune disorders with or without autoantibodies and/or a positive test for antibodies. Comorbidities included: arterial thrombotic events, venous thrombotic events, diabetes mellitus (DM; either self-reported and/or physician-based diagnosis and/or requiring pharmacologic treatment), hypertension, and osteoporosis.

MRI scans of brain lesions were classified as (1) normal; (2) nonspecific, described as a small number of white matter lesions with no MS features; (3) MS-like, lesions in regions considered typical of MS (e.g. periventricular, juxtacortical, callosal, and infratentorial); and (4) NMO-like, when lesions surrounded the fourth ventricle, hypothalamus, or aqueduct, with or without small deep white matter lesions [17, 18]. Spinal cord MRI scans were evaluated for lesion length. The length of the spinal cord lesions were expressed in terms of the number of vertebral segments. Laboratory tests of CSF included oligoclonal banding, cell counts, and protein concentrations.

### Ethical standard

This research was approved by the ethics committee of the Third Affiliated Hospital of Sun Yat-Sen University. All participants involved in this study provided written informed consent.

### Statistical analysis

All statistical analyses were performed using Statistical Program for Social Sciences (SPSS) statistical software (version 17.0; Chicago, IL, USA). Categorical variables were compared using Fisher’s exact test. Non-categorical variables were compared using the Mann–Whitney U test. Survival was estimated according to the Kaplan-Meier method. The log-rank test was used to compare the survival analysis between AQP4-seropositiveLO- and EONMOSD patients. Factors influencing outcomes were assessed independently by univariate binary logistic regressions (poor outcome defined as EDSS ≥ 4). Factors associated with a poor outcome were included in conditioned linear logistic analysis with binary distribution. Two-sided p values < 0.05 were considered significant.

## Results

We identified thirty AQP4-seropositive LONMOSD patients (Table 1). The median age at onset was 57.5 years (range 50–70), and 26 were female (86.7 %), similar to the early onset group (90 %). Attack onset occurred with similar frequencies between ON and LETM. Compared with AQP4-seropositive EONMOSD, interval between (first) ON and LETM and time from first symptoms to diagnosis of NMO were longer in the late-onset group (median 13 vs. 4 months, *p* < 0.05; median 17 vs. 7 months, *p* < 0.05). A higher proportion of comorbidities were displayed (66.7 vs. 26.7 %; *p* < 0.05), in which hypertension was displayed most frequently (26.7 vs. 3.3 %; *p* < 0.05). Other comorbidity-related variables were also observed in a higher proportion in late onset patients, although no significant differences were found. NMO-like lesions in the brain were observed less frequently (10.7 vs. 41.6 %; *p* < 0.05) in late-onset patients (Table 2). More non-specific lesions (53.6 vs. 29 %; *p* = 0.067) were also noted in this group, although no significant difference was found. No significant differences between groups were found in onset episode, monophasic form, first inter-attack interval, total attacks, ARR, coexisting autoimmunity, immunosuppressors, number of spinal cord MRI or cerebrospinal fluid results.

**Table 1:** Comparison of clinical features in patients with late-onset and early-onset AQP4-seropositive NMO/NMOSD

Feature	Late-onset AQP4-Ab Positive (*n* = 30)	Early-onset AQP4-Ab Positive (*n* = 30)	*p-*value
Total disease duration, months	24 (13–120)	25.5 (13–122)	0.958
Follow-up, median, mo	16 (12–84)	19 (12–88)	0.45
Female	26 (86.7)	27 (90)	0.5
Age at onset, median, y	57.5 (50–70)	31 (13–49)	<0.001
Onset episode, No.			
ON only	13 (43.3)	13 (43.3)	0.603
TM only	15 (50)	13 (43.3)	0.398
ON + TM	1 (3)	3 (10)	0.306
Brain/brainstem	0	0	-
Mixed e.g. ON + brain	1 (3)	1 (3)	0.754
Median first interattack interval, months	7.5 (1–34)	6 (0.5–56)	0.58
Median interval between first ON and LETM, months	13 (0–51)	4 (0–54)	0.022
Median time from first symptoms to diagnosis of NMO	17 (0.5–52)	7 (0.5–54)	0.009
Monophasic	2 (6.6)	2 (6.6)	0.694
Total n of attacks	2.5 (1–11)	3 (1–7)	0.571
ARR	1.37 (0.5–2.9)	1.50 (0.5–2.99)	0.398
Coexisting autoimmunity, No.	11 (36.7)	7 (23.3)	0.20
Coexisting autoimmune disorders^a^	4	2	0.57
Autoantibodies (number of positive tested/number tested)	7	5	0.57
Comorbidities, No.	20 (66.7)	8 (26.7)	0.002
Arterial thrombotic events	1 (3)	0 (0)	0.5
Venous thrombotic events	2 (6.7)	1 (3)	0.5
Diabetes mellitus	5 (16.7)	3 (10)	0.35
Hypertension	8 (26.7)	1 (3)	0.01
Osteoporosis	5 (16.7)	2 (6.6)	0.21
^b^Immunosuppressors	27 (90)	28 (93.3)	0.5
Corticosteroids	13	16	0.35
Azathioprine + corticosteroids	12	9	0.25
Tacrolimus + corticosteroids	2	3	0.52
Nadir EDSS score	6.75 (3–8.5)	5 (3–8)	0.007
Time to EDSS 4	8 (0–39)	13.5 (0–54)	0.026
EDSS score at last visit	5.25 (1.5–10)	4 (0.5–7.5)	0.002

Values are presented as frequencies (percentages) and medians (minimum-maximum)

*ON* optic neuritis, *LETM* longitudinally extensive transverse myelitis, *ARR* annualized relapse rate, *EDSS* Expanded Disability Status Scale

^a^Coexisting autoimmune disorders with or without autoantibodies included: Late-onset AQP4-Ab positive group: Sjögren syndrome (3 cases), Rheumatoid arthritis (1 case); early-onset AQP4-Ab positive group: Sjögren syndrome (1 case), Graves’ disease (1 case)

^b^Autoantibodies (number of positive tested/number tested) included: Late-onset AQP4-Ab Positive group: anti-deoxyribo-nuclease B antibody (1 case), anti-nuclear antibody (2 cases), anti-histone antibody (2 cases), anti-keratin antibody (1 case), anti-cyclic citrullinated peptide antibody (1 case); Early-onset AQP4-Ab Positive group: anti-double-stranded DNA antibody (1 case), anti-nuclear antibody (2 cases), anti-histone antibody (2 cases)

**Table 2:** MRI and CSF findings in patients with late-onset and early-onset AQP4-seropositive NMO/NMOSD

Feature	Late-onset AQP4-Ab Positive	Early-onset AQP4-Ab Positive	*p* value
Brain MRI, *n*	28 (93.3)	24 (80)	
Normal	4 (14.3)	5 (21)	0.398
Nonspecific	15 (53.6)	7 (29)	0.067
MS-like	1 (3.6)	3 (12.5)	0.249
NMO-like	3 (10.7)	10 (41.6)	0.012
Spinal cord MRI, *n*	28 (93.3)	27 (90)	
Length of spinal lesion, VBs, median	6 (3–16)	5 (0–19)	0.914
CSF analysis			
CSF pleocytosis, WBC count ≥10/μL, n/total	5/24 (20.8)	6/24 (25)	0.5
Elevated CSF protein, >0.6 g/L, *n*/total	4/24 (16.7)	3/24 (12.5)	0.45
Oligoclonal bands positivity, *n*/total	1/20 (5)	2/20 (10)	0.46

Values are represented as frequencies (percentages; % of those examined) and medians (minimum–maximum)

*MS* multiple sclerosis, *NMO* neuromyelitis optica, *VB* vertebral body, *CSF* cerebrospinal fluid, *WBC* white blood cells

**Fig. 1:**
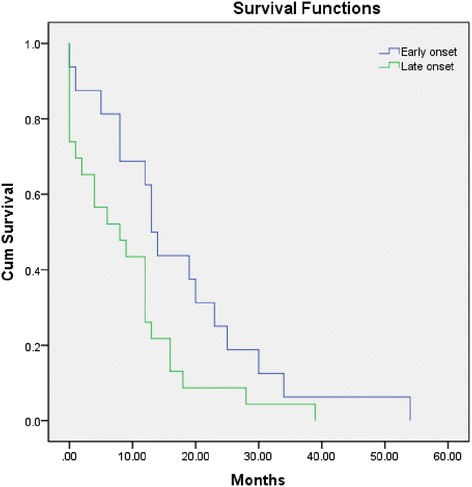
Time to the assignment of Expanded Disability Status Scale 4 in AQP4-positive neuromyelitis optica spectrum disorder patients with early-onset vs. late-onset

Compared with AQP4-seropositive EONMOSD, the severe EDSS scores in nadir (6.75 vs. 5; *p* < 0.05) were higher in the late-onset group. The median time to EDSS 4 in AQP4-positive LONMOSD was significantly less than in AQP4-positive EONMOSD (8 vs. 13.5 months; *p* < 0.05), as illustrated in Fig. 1. At the end of the follow-up, the EDSS score was more severe (median 5.25 vs. 4; *p* < 0.05) in the late-onset group compared with the early-onset group. Only one patient from the late-onset group died soon after hospitalization during the third attack. Death in this patient was secondary to extensive myelitis with pulmonary infection and respiratory failure. Finally, no significant prognostic factors were identified using univariate binary logistic regression; thus, conditioned linear logistic analysis was not performed.

## Discussion

Although the influences of differences in onset age in NMOSD have been documented in a number of previous reports, there are no systematic studies comparing late-onset to early-onset AQP4-seropositive NMOSD. This study showed that AQP4-seropositive LONMOSD was often characterized by severe attacks and resulted in disability within a shorter time, compared with AQP4-seropositive EONMOSD. Generally, our results do not support the hypothesis that patients with AQP4-seropositive LONMOSD show lower symptom severity, as has been reported in other autoimmune disorders [10, 11]. We also identified a number of distinct disease characteristics assumed to be age-related, such as longer interval between ON and LETM and time from first symptoms to diagnosis, more concurrent commodities, less NMO-like lesions and higher tendencies towards non-specific lesions. Finally, this study suggested that initial LETM may not necessarily be predominant among symptoms, as has been previously reported [6, 9, 12].

In our study, late-onset patients were particularly severe, with a higher disability rating after follow-up, in line with previous Caucasian and Japanese cohorts [6, 12]. However, the conclusions from the previous Caucasian study were limited by failing to set an early-onset control and by including AQP4-seronegative patients [12]. The creation of a Japanese cohort was an attempt to compare different clinical characteristics at different ages in AQP4-positive NMOSD patients; however, this comparison only occurred in a subanalysis, and therefore, a mere two difference variables (gender, lesion of first attack) were discussed [6]. In contrast to the study of the Japanese cohort, in which no information was given about total disease duration for either type of case (late onset) or controls (early onset), we matched our cases and controls for disease duration, which makes our observations even stronger.

It is reasonable that older patients are more susceptible to disability. During aging, there is a substantial decline in the ability to resist immune and inflammatory responses and a corresponding decline in the generation of robust protective immune responses, leading to a deficient anti-inflammatory process [19, 20]. It is also possible that late-onset patients show a less positive response to immunosuppressive therapy and experience more side effects and, at the same time, present with more frequent and severe comorbidities. Unfortunately, no significant prognostic factors were identified in the univariate analysis. This difficulty in identifying prognostic factors also occurred in a previous study [21]. Very few prognostic factors for poor outcomes have been identified in similar studies; among the few that have, a later onset age seems to be the most important variable [9, 12]. These scarce prognostic findings may partly reflect the different treatments, responses and unpredictable courses inherent in the disease, and it should be a call to the NMO community to establish a better cooperation model for the treatment of this late onset group.

Interestingly, we found longer interval between ON and LETM in late-onset patients, which has not been reported before. This observation may reveal differential age-dependent anatomical susceptibilities or age-related decreased susceptibility to AQP4 antibody accessibility from one target organ to another organ (ON to LETM, vice versa). Considering the severity of the pathology in each anatomical attack, the delay interval between ON and LETM emphasizes the importance of identifying early predictive factors for developing NMO from NMOSD in future research. Additionally, we observed that the time from first symptoms to diagnosis of NMO was longer than early-onset. This may partly be due to longer interval between ON and LETM per se. However, the possibility of an unusual presentation of late-onset patients must be kept in mind. For example, some AQP4-positive LONMOSD patients presented a clinical picture complicated by other commodities, higher tendencies towards non-specific lesions, and fewer classic brain lesions manifestations. This may tend to result in diagnostic delay.

Unlike previous reports indicating a predominance of LETM in NMOSD [6, 9, 12], we did not detect significant exhibition of LETM as the initial symptom in late-onset patients. Notably, our study cannot be directly compared with previous results, such as the Caucasian study describe above [12]. In fact, the frequency of exhibition of LETM in late-onset AQP4-seropositive patients was not conspicuous (44.4 %) in the Caucasian study, according to their subgroup analysis [12]. Additionally, the conclusions in previous studies that higher rates of myelitis observed in the UK and Japan were somewhat limited by failing to include an ethnically homogeneous population [9]. In another Japanese cohort, the chosen threshold cutoff age (60 years) differed from the cut-offs used in (other) NMO, multiple sclerosis and autoimmune disease research [6]. Another explanation for these differing symptom reports could be due to different AQP4-Abs tests and incomplete case ascertainment (e.g. a patient with minor ON may not be willing to participate in an AQP4-Abs test). Hence, the frequency of AQP4-seropositiveON could have been underestimated in some areas. This will tend to lead to higher frequencies of initial myelitis when calculating the proportion of initial symptom in NMOSD. Additionally, ethnic influence should still be considered as a potential explanation for this difference. A population-based study may improve conclusions about the prevalence of initial myelitis in these patients.

Concurrent underlying comorbidities increase the risk of complications in the elderly. Accordingly, this will require consideration of a wide differential diagnosis. As expected, hypertension was more frequent among late-onset patients, either alone or in addition to a higher prevalence of other comorbidities (Table 1). These processes are likely to be age related rather than NMOSD related, but they likely exert an additive or even a synergistic effect on morbidity and disability. Furthermore, the management of AQP4-seropositive LONMOSD is complicated by more common occurrence of DM and osteoporosis in these patients, limiting the use of corticosteroid treatment.

Our results revealed a high frequency of brain lesions (sum of non-specific, MS-like, and NMO-like), in line with previous reports [5, 6, 12]. However, unlike early-onset patients, we found that these NMO-like lesions were less often in AQP4-seropositive LONMOSD. This result may be a reflection of the intensity of the inflammatory process or a high level of autoimmune activity in early-onset patients (as opposed to late onset). Furthermore, we noted a tendency towards more frequent, non-specific lesions in AQP4-seropositive LONMOSD, partly reflecting the influence of these age-related comorbidities (e.g. heavier atherosclerotic burden). Taken together, these mixed neuroimaging characteristics of NMOSD may pose a greater challenge for differential diagnosis.

Our study had some limitations. First, owing to the retrospective design of this study, selection bias may have limited the interpretation of our results. The predominant recruitment of patients in our center may have led to benign types of patients being underrepresented, because potential mild types may fail to reach hospital attention. The detailed analysis of comorbidities and treatment effects in this study was also limited by its retrospective nature. Second, the low proportion of deaths compared with other studies may have been due to selection bias or to the relatively short follow-up period in our study (median 16 and 19 months in each group), limiting its interpretation. Third, our cohort could not use the updated version of diagnostic criteria for AQP4-seropositive LONMOSD, which was published recently, due to the earlier performance of this study [22]. However, we emphasized that this proposed diagnostic criteria is appropriate for our patients, whose diagnoses were based on the classic ON and/or LETM involvement, positive results of AQP4-IgG, and exclusion of alternative diagnoses at a center with well-established expertise in the NMOSD. Last but not least, we did not observe the first onset with more extensive CNS involvement compatible to the revised criteria other than ON and/or LETM involvement, for example, area postrema, other brainstem, diencephalon, or cerebra [22]. Although these unreported results may partly reflect the disease state of LONMOSD itself, it is also possible that future larger studies will led to recognition of more extensive manifestations using the revised criteria [22, 23].

Despite these limitations, our data extends our knowledge of the clinical, diagnostic and prognostic impacts of this rare yet often devastating condition. Our study also provides some explanation for the possible differences in NMOSD between younger and older onset cases and indicates some are related to NMOSD, and some are not related to NMOSD (e.g. comorbidities).

## Conclusion and implication

This study provides an overview of the clinical and paraclinical features of AQP4-seropositive LONMOSD patients in China and demonstrates a number of distinct disease characteristics in early vs. late onset. Our findings have several implications. In patients ≥50 years old, AQP4-seropositive NMOSD is dominant in females and occurs with similar frequencies and onset episodes; however, older patients are more susceptible to disability. Diagnoses delay may occur more frequently and diagnoses should be made with a wider differentialdiagnosis space due to a number of novel distinct disease characteristics in late onset, such as fewer NMO-like lesions in the presence of more non-specific lesions and more concurrent commodities. The higher burden of comorbidities may warrant a modified approach of treatment. Because there are no reliable prognostic biomarkers in AQP4-seropositive LONMOSD currently, it is difficult to predict outcomes or clinical courses at any stage in this group. Therefore, attack-prevention therapies should be initiated as early as possible, regardless of the initial presentation, course of disease, or degree of disability at the time of diagnosis.
